# The Reduced Nitridogermanates(III) Ca_6_[Ge_2_N_6_] and Sr_6_[Ge_2_N_6_] with Ge−Ge Bonds

**DOI:** 10.1002/anie.202017270

**Published:** 2021-02-18

**Authors:** Lukas Link, Manisha Pathak, Franziska Jach, Primoz Koželj, Alim Ormeci, Peter Höhn, Rainer Niewa

**Affiliations:** ^1^ Institute of Inorganic Chemistry University of Stuttgart Pfaffenwaldring 55 70569 Stuttgart Germany; ^2^ Chemical Metals Science Max Planck Institute for Chemical Physics of Solids Nöthnitzer Str. 40 01187 Dresden Germany; ^3^ Faculty of Chemistry and Food Chemistry Technische Universität Dresden 01062 Dresden Germany

**Keywords:** germanium, nitrides, nitridogermanates, sodium flux

## Abstract

The first nitridogermanates(III) Ca_6_[Ge_2_N_6_] and Sr_6_[Ge_2_N_6_] were synthesized from sodium flux and structurally characterized by powder and single crystal X‐ray diffraction, respectively. They crystallize isostructurally to each other and homeotypic to Ca_6_[Cr_2_N_6_]H in space group *R* $\bar{3}$ . They feature unprecedented, mutually isolated, ethane‐like [Ge^III^_2_N_6_]^12−^ anions in a staggered conformation. The compounds are semiconductors according to resistivity measurements and electronic structure calculations, yielding band gaps of 1.1 eV for Ca_6_[Ge_2_N_6_] and 0.2 eV for Sr_6_[Ge_2_N_6_].

## Graphical abstract

The nitridogermanates(III), Ca_6_[Ge_2_N_6_] and Sr_6_[Ge_2_N_6_], contain the hitherto unknown molecular anion [Ge_2_N_6_]^12−^ featuring a Ge−Ge‐bond and thus represent the first members of a novel class of compounds virtually unknown in the nitride chemistry of tetrel elements.

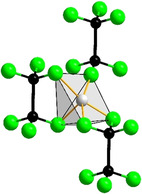

## Introduction

The binary nitrides of the heavier tetrels silicon, germanium, tin and lead show a clear trend with respect to the stability of the tetrel‐nitrogen bond. Silicon nitride Si_3_N_4_ is a well‐known, highly inert compound that undergoes significant thermal decomposition only at temperatures above 1500 °C.^[1]^ It has a large number of industrial and specialty applications due to its high hardness and thermal stability. Germanium nitride Ge_3_N_4_ is still a rather stable nitride, however, it decomposes around 600 °C in vacuum.^[2]^ The crystal structures of the low‐pressure modifications of both silicon and germanium nitride consist of three‐dimensional networks of corner‐sharing SiN_4_ or GeN_4_ tetrahedra. There also exists a high‐pressure spinel modification of both,[^3^, ^4^] in which two thirds of the tetrel atoms are coordinated octahedrally by nitrogen. Tin nitride Sn_3_N_4_ was so far exclusively observed in this modification. It is obtainable at atmospheric pressure, but starts to decompose around 300 °C.^[5]^ Lead nitride, to the best of our knowledge, has not been shown to exist as of yet.

Due to the intermediate stability of the germanium‐nitrogen bond, Ge is unique among the group 14 elements when it comes to ternary nitrides. As discussed above, the heavier elements tin and lead are very reluctant to form bonds with nitrogen at all, with NaSn^II^N currently being the only known ternary nitridostannate.^[6]^ Silicon on the other hand is so strongly attracted to nitrogen that [Si^IV^N_4_]^8−^ tetrahedra and their condensates are usually the only structural units found in nitridosilicates.^[7]^ Exceptions from this rule include the (oxido)nitridosilicate Ce_16_(Si_15_O_6_N_32_) containing SiN_6_ octahedra and particularly SrSi_6_N_8_ and BaSi_6_N_8_, which feature Si^III^−Si^III^ bonds next to dominating vertex‐sharing Si^IV^N_4_ tetrahedra.[^8^, ^9^, ^10^] Nitridogermanates exhibit a much richer structural chemistry than known for nitridosilicates or ‐stannates. Building blocks can include bent [Ge^II^N_2_]^4−^ anions,[^11^, ^12^, ^13^] trigonal planar [Ge^IV^N_3_]^5−^ units,^[14]^ as well as [Ge^IV^N_4_]^8−^ tetrahedra.[^11^, ^15^] As with the silicates, these latter tetrahedra can condense via corner‐sharing to yield extended units or via edge‐sharing, resulting in [Ge_2_^IV^N_6_]^10−^ units.^[16]^ Instances of nitridogermanate units coexisting with both isolated nitride ions and germanide Zintl ions have also been shown to exist.[^13^, ^17^, ^18^]

Here we present a hitherto unknown structural unit, the [Ge^III^_2_N_6_]^12−^ anion, featuring two germanium(III) atoms connected by a direct covalent bond and three nitride ligands each. In the title compounds, the [Ge^III^_2_N_6_]^12−^ anion is arranged in a staggered conformation, consistent with point group $\bar{3}$ , within the crystal structure arrangement it retains full $\bar{3}$ *m* (*D*_3*d*_) symmetry, even though the crystal structure as a whole does not. This anion is notably distinct from the [Ge^IV^_2_N_6_]^10−^ unit formed by two edge‐sharing tetrahedra found in Sr_5_[Ge_2_N_6_].^[16]^ Similar anionic units were revealed, for example, in a class of alkali metal chalcogenidotetrelates with the general formula *A*_6_[*Tt*_2_ *Ch*_6_] and *A*=Na, K, Cs; *Tt*=Si, Ge, Sn; *Ch*=S, Se, Te.[^19^, ^20^, ^21^, ^22^, ^23^] Each of those compounds features ethane‐analogue [*Tt*_2_ *Ch*_6_]^6−^ ions. Related compounds in the nitridometalate chemistry of transition metals include the homeotypic hydride Ca_6_[Cr_2_N_6_]H,^[24]^ as well as Sr_2_Li_6_[Mn_2_N_6_] and Ca_2_Li_6_[Mn_2_N_6_],[^25^, ^26^] which contain [Mn^IV^_2_N_6_]^10−^ anions in a similar rhombohedral packing motif.

## Results and Discussion

Dark grey single phase microcrystalline Ca_6_[Ge_2_N_6_] was obtained from calcium nitride Ca_3_N_2_, elemental Ge and sodium azide in a molar ratio *n*(Ca):*n*(Ge):*n*(N) of 3:1:3.7 in sealed tantalum ampoules at 800 °C. Sr_6_[Ge_2_N_6_] was synthesized by reacting strontium nitride Sr_2_N, germanium powder and sodium azide in a molar ratio *n*(Sr):*n*(Ge):*n*(N) of 3:1:3.5 in sealed niobium ampoules at 720 °C, using molten sodium flux. In each of three batches using this method, a small quantity of black Sr_6_[Ge_2_N_6_] single crystals was found, alongside mostly black, rod‐shaped Sr_2_[GeN_2_] crystals, yellow platelets of Sr_5_[Ge_2_N_6_] and red Sr_7_[GeN_6_] crystals.[^12^, ^15^, ^16^] In a later attempt to synthesize Sr_6_[Ge_2_N_6_], elemental germanium was replaced in the synthesis by germanium nitride Ge_3_N_4_ while keeping the overall nitrogen amount identical by reducing the sodium azide quantity. All other products resulted in similar proportions as before, but no Sr_6_[Ge_2_N_6_] was found in this batch. This suggests that Sr_6_[Ge_2_N_6_] might form as an intermediate during intercalation of strontium and concomitant nitridation of elemental germanium, retaining an isolated Ge−Ge bond. More detailed information on synthesis and crystal structure determinations can be found in the supporting information.

**Figure 1:**
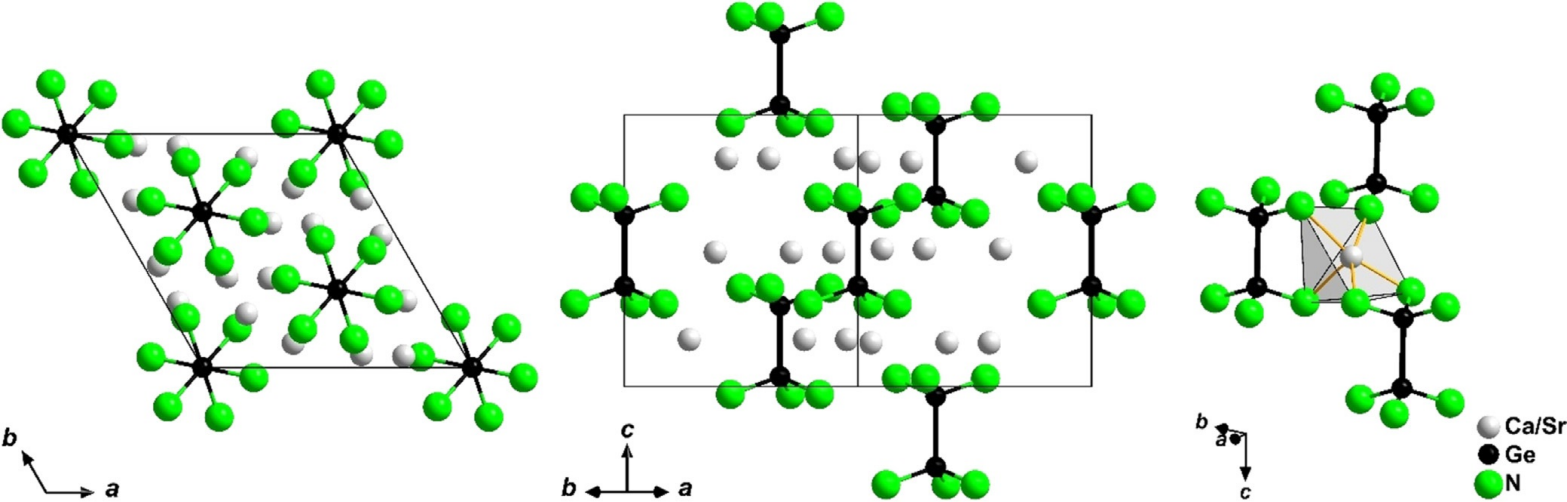
Two projections of the extended unit cell of the title compounds (left, center) and coordination of the alkaline earth metal ions (right).^[33]^

The title compounds crystallize homeotypic to Ca_6_[Cr_2_N_6_]H,^[24]^ which features ethane‐like [Cr_2_N_6_]^11−^ anions as well as hydride anions. The hydride ions are coordinated octahedrally by six calcium atoms. However, the site at (0, 0, 0), where hydride is found in Ca_6_[Cr_2_N_6_]H, is unoccupied in the title compounds. The distance from the vacant site to each neighboring cation amounts to 2.96 Å for Sr_6_[Ge_2_N_6_] and 2.85 Å for Ca_6_[Ge_2_N_6_], which would leave ample space for an anion such as N^3−^ or H^−^. However, no significant residual electron density was found in this position, which is consistent with the charge balance of the compounds leaving no room for additional charged constituents. As depicted in Figure 1, this arrangement leads to a distorted square pyramidal coordination for the alkaline earth metal ions, where a distorted octahedral coordination complemented by H^−^ was reported for Ca_6_[Cr_2_N_6_]H.

Table 1 lists the most characteristic bond distances and angles in the title compounds. Perhaps unexpectedly, the Ge−Ge bond distances are significantly longer in Sr_6_[Ge_2_N_6_] than in Ca_6_[Ge_2_N_6_]. Though the difference may seem rather large, both values are within the range of Ge−Ge single bond distances reported for similar germanium(III) compounds, for example, the alkali metal chalcogenidogermanates with Ge−Ge bond distances as short as 2.43 Å in Na_6_[Ge_2_Se_6_] or as long as 2.58 Å in K_6_[Ge_2_S_6_].[^22^, ^23^] Even longer distances are found in SrGe, a Zintl phase featuring infinite (Ge^2−^) chains with a bond length of 2.63 Å.^[27]^ Lastly, the distance between two germanium atoms bridged by two nitrogen atoms in Sr_5_[Ge^IV^_2_N_6_] is reported at 2.61 Å,^[16]^ which makes it only slightly longer than the Ge‐Ge distance in Sr_6_[Ge_2_N_6_].

**Table 1:** Selected distances (Å) and angles (deg.) found in the title compounds (*A*=Ca, Sr).^[33]^

	Ca_6_[Ge_2_N_6_]	Sr_6_[Ge_2_N_6_]
*d*_avg_(*A−*N) [Å]	2.515	2.666
*d*_min_(*A−*N) [Å]	2.409(12)	2.594(3)
*d*_max_(*A−*N) [Å]	2.728(13)	2.803(3)
*d*(Ge*−*Ge) [Å]	2.419(5)	2.595(1)
*d*(Ge*−*N) [Å]	1.891(18)	1.908(1)
*α*(Ge‐Ge‐N) [°]	108.7(4)	107.1(1)
*α*(N‐Ge‐N) [°]	110.3(7)	111.7(1)

The Ge−N bond distances found for the title compounds are similar to those of nitridogermanates(IV) with a tetrahedral coordination of germanium, such as Sr_7_[GeN_4_]N_2_ with *d*(Ge‐N)=1.92 Å,^[15]^ Sr_5_[Ge_2_N_6_] with 1.84 Å< *d*(Ge‐N) <1.99 Å,^[16]^ and Ca_4_[GeN_4_] with 1.89 Å< *d*(Ge‐N) <1.95 Å.^[11]^ Nitridogermanates(II) generally contain slightly shorter Ge‐N distances, typically ranging between 1.84 Å and 1.89 Å,[^13^, ^17^, ^18^] with Sr_3_Ge[GeN_2_] being the only outlier with a reported Ge‐N distance of 1.94 Å.^[12]^

**Figure 2:**
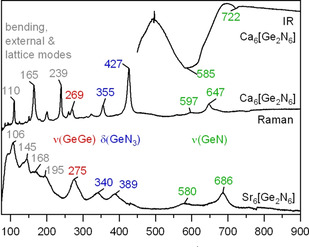
IR and Raman spectra of microcrystalline powders of Ca_6_[Ge_2_N_6_] (top), and a crystal of Sr_6_[Ge_2_N_6_] (bottom).

Figure 2 depicts IR and Raman spectra of Ca_6_[Ge_2_N_6_] and Sr_6_[Ge_2_N_6_]. In accordance to point group *D*_3*d*_ of the ethane‐like anion, three clearly distinguishable regions are visible: the ν(GeGe) mode (A_1g_), two Raman active modes for δ(GeN_3_) (A_1g_, E_g_), and four ν(Ge‐N) modes in IR (A_2u_, E_u_) and Raman (A_1g_, E_g_) spectra.^[28]^ The Ge‐Ge stretching mode is close to 300 cm^−1^ which was reported for elemental germanium,^[29]^ despite the different bond lengths. Deformation modes δ(GeN_3_) agree well with those of various Ge_3_N_4_ modifications.^[30]^ In contrast, stretching modes ν(GeN) are shifted to lower frequencies in comparison to Ge_3_N_4_. This corresponds well to the longer Ge−N bonds of terminal N atoms in [Ge_2_N_6_]^12−^ and shorter bonds for the Ge‐N‐Ge bridging unit in Ge_3_N_4_.^[31]^ A similar situation is observed for other isolated molecular anions such as [GeN_2_]^4−^ and [GeN_4_]^8−^ whose ν(GeN) modes accord well with the title compounds (Figure S2, Table S5).

Measurements of the temperature dependence of the magnetization were performed via SQUID magnetometry for magnetic fields up to 7 T and temperatures between 1.8 and 350 K. They indicate that Ca_6_[Ge_2_N_6_] is diamagnetic—see the supplementary material for details—which is consistent with what can be expected from the constituent elements and bonding. Electrical resistivity measurements were performed with a sapphire pressure cell from about 150 K to room temperature. Measurements of the resistivity of a Ca_6_[Ge_2_N_6_] sample exhibit activated semiconducting‐like behavior consistent with the sample being diamagnetic.

**Figure 3:**
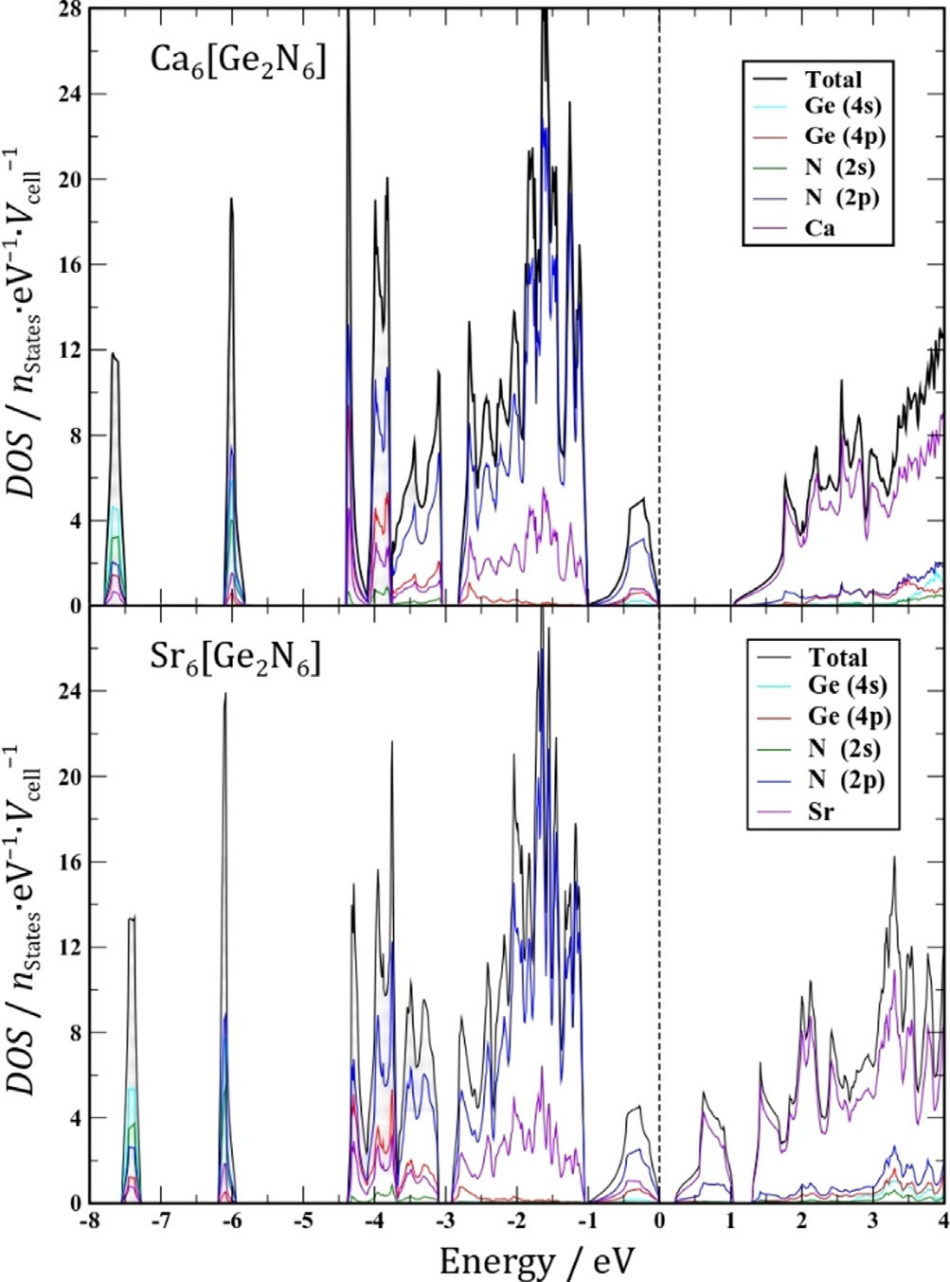
Total and partial electronic density of states computed for Ca_6_[Ge_2_N_6_] and Sr_6_[Ge_2_N_6_]. The band gaps are 1.1 eV and 0.2 eV, respectively.

The electronic structure calculations show that both Ca_6_[Ge_2_N_6_] and Sr_6_[Ge_2_N_6_] are semiconductors with band gaps of 1.1 and 0.2 eV, respectively (see Figure 3). The densities of states (DOS) of both compounds are very similar throughout the whole valence band region. There are two very narrow bands at around −7.5 and −6.0 eV, respectively. The largest contributions to the former are from germanium and nitrogen *s* states, while N 2*p* is the largest contributor to the latter ahead of the *s* states. The higher energy regions are structured into three segments with the following approximate boundaries: [−4.4, −3.1], [−2.9, −1.0] and [−1.0, 0.0] eV (top of the valence band is set to 0 eV). The largest contributions come from the N 2*p* states, with Ca or Sr contributions (mainly from the 3*d* or 4*d* states, respectively) becoming significant in the last two segments. Most of the Ge 4*p* contributions are in the segment ranging from −4.4 to −3.1 eV. In both phases, the alkaline earth metal elements dominate the conduction bands.

**Figure 4:**
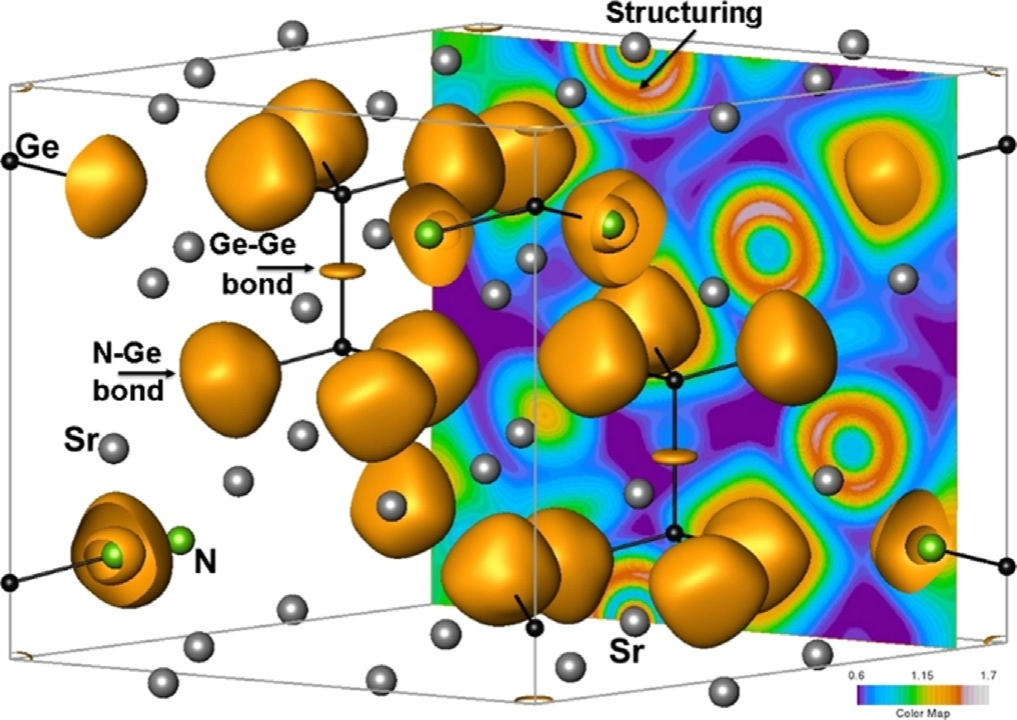
The ELI distribution in the hexagonal unit cell of Sr_6_[Ge_2_N_6_] with the isosurface value of 1.38. The two‐dimensional plot of ELI in the *(a*,*c)* plane highlights the structuring of the penultimate shell (*n=*4) of the Sr atoms.

**Figure 5:**
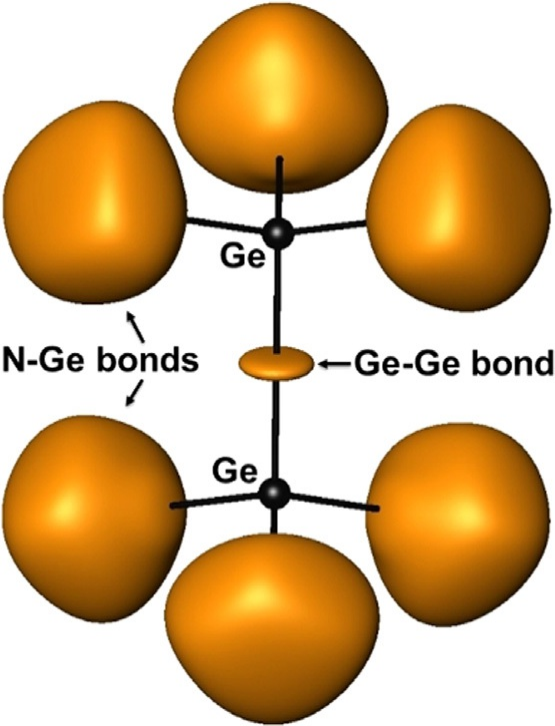
The ELI distribution with isosurface value of 1.385 around the [Ge_2_N_6_]^12−^ ion displays how the Ge−Ge and N−Ge bonds are organized spatially. The N atoms are located inside the isosurface regions representing the N−Ge bonds.

The similarities between the two compounds extend to their effective charges and chemical bonding situation, as well. The effective charges computed based on the QTAIM approach yield positive charges amounting to 1.37 for calcium, 1.36 for strontium and 1.14 for germanium, in both cases. Hence, nitrogen atoms hold an effective charge of −1.75 in Ca_6_[Ge_2_N_6_] and −1.74 in Sr_6_[Ge_2_N_6_]. These high effective charge values imply significant ionic interaction contribution to the cohesion in the title compounds. The topological analysis of the ELI reveals the chemical bonding situation in the hexagonal unit cell as depicted in Figure 4. The organization of the two types of bonds in the [Ge_2_N_6_]^12−^ ion is shown in Figure 5. The two‐center Ge−Ge bond has an electron population of *n*_b_^Ge‐Ge^=2.72 and 2.70 in Ca_6_[Ge_2_N_6_] and Sr_6_[Ge_2_N_6_], respectively. The volume of the Ge−Ge bond basin, *V*_b_^Ge‐Ge^, in Sr_6_[Ge_2_N_6_] (13.67 Å^3^) is larger than that in Ca_6_[Ge_2_N_6_] (11.91 Å^3^), however when expressed as a fraction of the unit cell volume, these values become comparable, 1.73 % versus 1.75 %, respectively. Additionally, if we define density of bonding electrons as *ρ*_b_^Ge‐Ge^=*n*_b_^Ge‐Ge^/ *V*_b_^Ge‐Ge^, then we find that *ρ*_b_^Ge‐Ge^ in Ca_6_[Ge_2_N_6_] (0.228 e^−^ Å^−3^) is higher than that in Sr_6_[Ge_2_N_6_] (0.198 e^−^ Å^−3^) reflecting, essentially, the effect of difference in unit cell volumes rather than difference in Ge−Ge bond distances. The other bond is a highly polar Ge−N bond at which each of the five alkaline earth metal atoms coordinating to the nitrogen atom participates at a level less than 1 %. There are six such bonds per formula unit (alternatively one per N atom). The electron population of the bond basin is 7.50 in Ca_6_[Ge_2_N_6_] and 7.48 in Sr_6_[Ge_2_N_6_] with the largest contributions coming from the N atoms, at 6.62 and 6.59 electrons, respectively. Ge contributions are 0.57 and 0.58 electrons, while individual alkaline earth metal atoms provide only about 0.06 electrons so that total Ca or Sr contribution amounts to 0.30 and 0.31, respectively.

ELI valence region attractors and the associated basins involve the genuine valence electrons of the atoms. The electrons occupying the Ca 3*d* or Sr 4*d* states belong to the penultimate shell (principal quantum number equal to 3 and 4, respectively). The participation of these electrons in the chemical bonding can be concluded if the distribution of the ELI in the penultimate shell deviates from spherical symmetry, a situation referred to as structuring.^[32]^ Based on the electronic DOS (Figure 3), the occupancies of the Ca 3*d* and Sr 4*d* states are computed as 0.54 and 0.65 electrons, respectively. The implied participation of these electrons in chemical bonding is identified in Figure 4 for the exemplary case of Sr_6_[Ge_2_N_6_].

## Conclusion

Ca_6_[Ge_2_N_6_] and Sr_6_[Ge_2_N_6_] are two new compounds containing the hitherto unknown molecular anion [Ge_2_N_6_]^12−^ and particularly the first nitridogermanates(III), a novel class of compounds virtually unknown in nitride chemistry of the tetrels. Both compounds crystallize homeotypic to Ca_6_[Cr_2_N_6_]H, although the hydride site is left unoccupied. Like the majority of nitridogermanates reported so far, the anions in Ca_6_[Ge_2_N_6_] and Sr_6_[Ge_2_N_6_] are isolated from each other and do not condense to form chains, layers or three‐dimensional networks, as would be very typical for the related nitridosilicates.

## Conflict of interest

The authors declare no conflict of interest.

## Supporting information

As a service to our authors and readers, this journal provides supporting information supplied by the authors. Such materials are peer reviewed and may be re‐organized for online delivery, but are not copy‐edited or typeset. Technical support issues arising from supporting information (other than missing files) should be addressed to the authors.

Supplementary
