# Supplementary material for: The Reduced Nitridogermanates(III) Ca6[Ge2N6] and Sr6[Ge2N6] with Ge−Ge Bonds
Source: Angew Chem Int Ed Engl. 2021 Feb 18;60(14):7691–6. doi: 10.1002/anie.202017270 (PMC8048604; doi:10.1002/anie.202017270)
Supplement: Supplementary file 1 — Supplementary [file ANIE-60-7691-s001.pdf]

Angewandte Chemie
Eine Zeitschrift der Gesellschaft Deutscher Chemiker

Supporting Information

The Reduced Nitridogermanates(III) $Ca_6[Ge_2N_6]$ and $Sr_6[Ge_2N_6]$ with Ge−Ge Bonds

L. Link, M. Pathak, F. Jach, P. Koželj, A. Ormeci, P. Höhn, and R. Niewa**

anie_202017270_sm_miscellaneous_information.pdf

Table of Contents

Table of Contents 2
Experimental Procedures 3
Preparation 3
X-ray diffraction 3
Figure S1. Rietveld fit of $Ca_6[Ge_2N_6]$ powder X-ray diffraction data (above) together with the difference curve (below). Gray areas refer to regions excluded from refinement due to unidentified impurities. 3
Table S1. Crystallographic data for the title compounds. 4
Table S2. Atom sites and isotropic displacement factors for $Ca_6[Ge_2N_6]$. 4
Table S3. Atom sites and isotropic displacement factors for $Sr_6[Ge_2N_6]$. 4
Table S4. Anisotropic displacement factors for $Sr_6[Ge_2N_6]$. 5
Vibrational spectroscopy 5
Magnetic properties investigation 6
Figure S2. Molar magnetic susceptibility, only 10000 Oe and 70000 Oe shown. Considering the high-temperature part, it is obvious that $Ca_6[Ge_2N_6]$ is diamagnetic. 6
Electronic conductivity investigations 6
Figure S3. Electrical resistivity of $Ca_6[Ge_2N_6]$ as measured in a sapphire pressure cell. See text for discussion. Below 220 K, the resistivity is not shown as it becomes too large to be measured properly with our experimental set-up. 7
Electronic structure calculations 7
References 8
Author Contributions 8

Experimental Procedures

Preparation

All experimental steps were carried out under an inert atmosphere in a glove box (MBraun, ≤ 1 ppm O_2) due to the high reactivity of both products and educts towards oxygen and moisture.

Calcium and strontium nitride, Ca_3N_2 and Sr_2N, were prepared by placing a tantalum crucible with alkaline earth metal (dentritic, Sigma-Aldrich, 99.99 %) in a quartz tube and heating for 8 hours at 550 °C under nitrogen atmosphere (Alphagaz, 99.999 %). The product was crushed and re-reacted for another 8 hours.

Synthesis of dark grey $Ca_6[Ge_2N_6]$ was performed from Ca_3N_2 and Ge in molar ratio n(Ca) : n(Ge) : n(N) of 3 : 1 : 3.7 in sealed tantalum ampoules at 750 °C to 800 °C for 36 h to 48 h (heating/cooling rate 100 °C · h^{-1}) using NaN_3 as a source of N_2. Several heating/grinding/repelletizing cycles with additional NaN_3 were needed to obtain a single phase sample and to remove Ca_3GeN and $Ca_2[GeN_2]$ impurities. After the reaction, excess Na was removed under dynamic vacuum at 300 ºC.

Black single crystals of $Sr_6[Ge_2N_6]$ were grown from strontium nitride, germanium powder and sodium azide NaN_3 in sealed niobium ampoules using sodium metal as a flux. The ampoules were heated in a quartz tube under argon at 720 °C for three days, after which they were allowed to naturally cool to room temperature. The ampoules were cut open and the sodium was removed by extraction with liquid ammonia. Samples contained very few trigonal antiprismatic $Sr_6[Ge_2N_6]$ crystals and consisted mostly of black, rod-shaped $Sr_2[GeN_2]$ [1] crystals, yellow platelets of $Sr_5[Ge_2N_6]$ [2] and red $Sr_7[GeN_4]N_2$ [3] crystals.

Microcrystalline powders of $Ca_2[GeN_2]$,[4] $Sr_2[GeN_2]$,[1] $Ca_4[GeN_4]$,[4] $Sr_7[GeN_4]N_2$,[3] and Ge_3N_4 [5-6] were prepared as reference materials according to previously reported protocols.

X-ray diffraction

Lattice and atomic parameters of $Ca_6[Ge_2N_6]$ were refined with the Jana2006 program package [7] employing pseudo-Voigt profile functions against laboratory powder X-ray diffraction data of finely ground powder samples collected on a Huber G670 imaging plate Guinier camera using a curved germanium (111) monochromator and Cu-Kα1 radiation in the range 10.3° ≤ 2θ ≤ 96° with an increment of 0.005° at 293(1) K. The powder samples were placed between Kapton foils to avoid degradation in air. The powder pattern depicted in Figure S1 showed small amounts of elemental sodium, which were included in the refinement, and small amounts of a hitherto unknown impurity phase, which were excluded from refinement.

Single crystals of $Sr_6[Ge_2N_6]$ with well-defined features were selected and sealed in glass capillaries and single crystal X-ray diffraction data was collected on a Bruker κ-CCD diffractometer using Mo-$K_α$ radiation. A numerical absorption correction was applied using X-SHAPE and the structure model was solved and refined using the SHELX software.[8-9] Graphical representations of the structure were created in Diamond.[10]

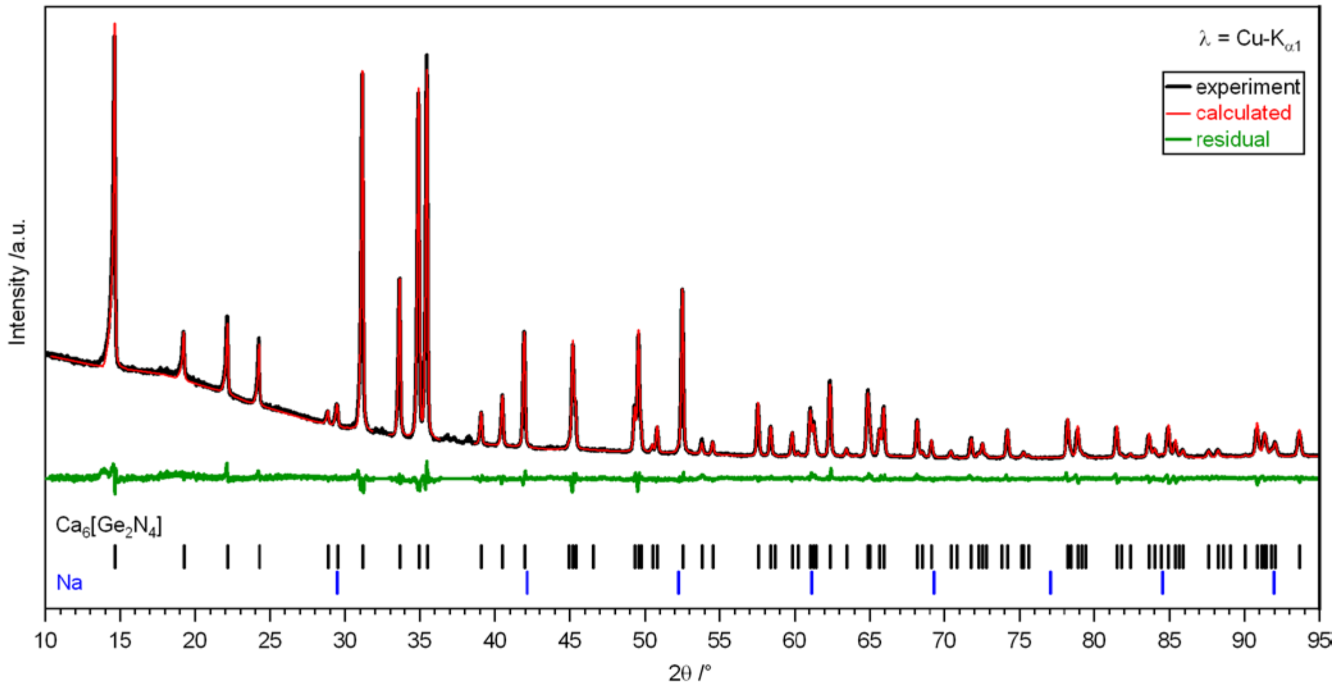

Figure S1. Rietveld fit of $Ca_6[Ge_2N_6]$ powder X-ray diffraction data (above) together with the difference curve (below). Solely black areas refer to regions excluded from refinement due to unidentified impurities.

Table S1. Crystallographic data for the title compounds.

Compound	$Ca_6[Ge_2N_6]$	$Sr_6[Ge_2N_6]$
Crystal system	Trigonal	
Space group	$R\overline{3}$ (No. 148)	
a / Å	9.2078(2)	9.6655(4)
c / Å	9.2679(3)	9.7757(4)
V / Å^3	680.50	790.91
Z	3	
Calculated density ρ / g·cm^{-3}	3.439	4.755
Absorption coefficient μ / mm^{-1}	37.75	35.68
Temperature T / K	293	
Wavelength / Å	1.54056	0.71073
F(000)	678	1002
hkl range		±12 each
$2\theta_{max}$	96°	55.6°
R_{int} / R_{σ}		5.66 % / 2.56 %
R_1 / wR_2		1.91 % / 3.87 %
wR_P / wR (all F_o)	3.11 % / 3.51 %	
GooF	1.62	1.098
Extinction coefficient		0.0019
Unique reflections measured		417
Parameters refined	14	23
Highest electron diff. peak	0.48 Å^{-3}	1.29 Å^{-3}
Deepest electron diff. hole	–0.42 Å^{-3}	–1.35 Å^{-3}

Table S2. Atom sites and isotropic displacement factors for $Ca_6[Ge_2N_6]$.

Atom	Site	x / a	y / b	z / c	U_{eq} / pm^2
Ca	18*f*	0.0484(5)	0.2777(4)	0.1727(4)	0.0156(15)
Ge	6*c*	0	0	0.3695(4)	0.0139(12)
N	18*f*	0.2129(14)	0.1686(15)	0.3042(12)	0.004(4)

Table S3. Atom sites and isotropic displacement factors for $Sr_6[Ge_2N_6]$.

Atom	Site	x / a	y / b	z / c	U_{eq} / pm^2
Sr	18*f*	0.05184(4)	0.27783(4)	0.16719(3)	0.0106(2)
Ge	6*c*	0	0	0.36725(6)	0.0099(2)
N	18*f*	0.2062(4)	0.1639(4)	0.3098(3)	0.0100(6)

Table S4. Anisotropic displacement factors for $Sr_6[Ge_2N_6]$.

Atom	U_{11} / pm^2	U_{22} / pm^2	U_{33} / pm^2	U_{23} / pm^2	U_{13} / pm^2	U_{12} / pm^2
Sr	0.0090(2)	0.0100(2)	0.0103 (2)	–0.0016(1)	0.0001(1)	0.0029(1)
Ge	0.0062(2)	U_{11}	0.172(4)	0	0	½ U_{11}
N	0.010(2)	0.007(1)	0.014(2)	–0.002(1)	0.000(1)	0.005(1)

Vibrational spectroscopy

Raman spectra of microcrystalline powders of $Ca_6[Ge_2N_6]$ and a crystal of $Sr_6[Ge_2N_6]$ were collected at room temperature using a Horiba LabRam HR Evolution spectrometer equipped with a Laser Quantum Ventus diode laser (532 nm). The spectral resolution was about 0.42 cm^{-1}. The samples were sealed in capillaries for protection against air and moisture. Infrared spectra of finely ground powder samples of $Ca_6[Ge_2N_6]$ and reference samples of $Ca_2[GeN_2]$, $Sr_2[GeN_2]$, $Ca_4[GeN_4]$, $Sr_7[GeN_4]N_2$, and Ge_3N_4 were recorded in Attenuated Total Reflectance (ATR) mode using a PerkinElmer UATR-Two FTIR spectrometer inside a glove box (spectral range 4000 − 450 cm^{-1}). No signals indicating moisture in the samples have been detected by either IR or Raman spectroscopy.

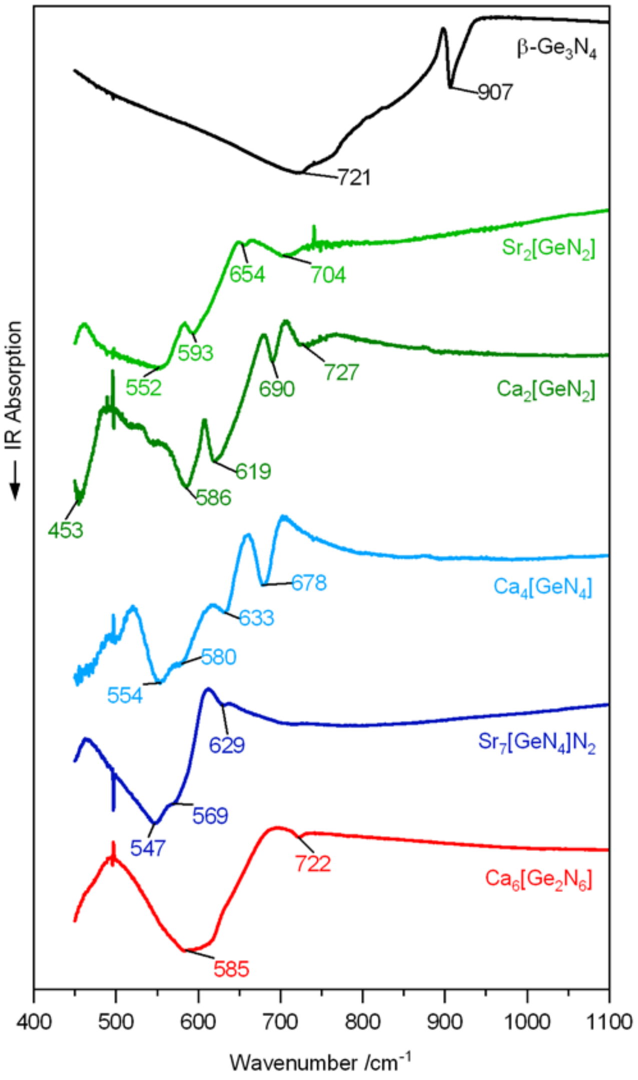

Figure S2. IR spectra of various Ge-N compounds. Modes between 550 and 950 cm^{-1} can be assigned to ν(GeN) stretching vibrations.

Table S5. Synopsis of bond lengths /Å and ν(GeN) stretching frequencies /cm^{-1} as observed in IR spectra (Figure S2) in germanium nitride compounds.

Compound	Ge−N distances [Å]	ν(GeN) stretching frequencies [cm^{-1}]	Reference
β-Ge_3N_4	1.83 – 1.85	721, 907	[5-6]
$Ca_2[GeN_2]$	1.859(4), 1.872(4)	552 – 704	[4], this work
$Sr_2[GeN_2]$	1.851(11), 1.877(9)	586 – 727	[1], this work
$Ca_4[GeN_4]$	1.889(2), 1.892(2), 1.906(2), 1.950(2)	554 – 678	[4], this work
$Sr_7[GeN_4]N_2$	1.922(4), 1.923(5)	547 – 629	[3], this work
$Ca_6[Ge_2N_6]$	1.891(18)	585 – 722	this work

Magnetic properties investigation

Magnetism measurements were performed on a Quantum Design on a MPMS XL-7 SQUID magnetometer between 1.8 and 350 K in external fields up to 7 T, see fig. S1. Before the measurements, the sample was sealed into a quartz tube so that it could be safely measured without exposing it to oxidation. As the magnetic moment of the quartz tube was premeasured, it was be subtracted from the measured moment.

Since it was observed that the high temperature part of the measured magnetizations shifts upwards with increasing field, it was concluded that the sample contains some ferromagnetic impurities. A simple Hondo-Wilson-like correction was applied to shift the curves back together.[11-12] The required shift corresponds to 0.005 wt.% of iron, which is a reasonable quantity attributed to either impurities in the chemicals or contamination during sample handling. The magnetism after correcting for ferromagnetic impurities shown in fig. S1 is diamagnetic with paramagnetic tail at low temperature. As $Ca_6[Ge_2N_6]$ does not contain elements for which localized magnetic moments would be expected, the diamagnetism (~ $-75\cdot10^{-6}$ emu/mol) is attributed to $Ca_6[Ge_2N_6]$, whereas the paramagnetic tail is just due to small amounts of unspecified impurities.

Calculation of the diamagnetic contribution of $Ca_6[Ge_2N_6]$ is difficult considering the limited data available. Values are listed for Ca^{2+} ($-8\cdot10^{-6}$ emu/mol, $-10.4\cdot10^{-6}$ emu/mol)[13-14] and N^{3-} ($-13\cdot10^{-6}$ emu/mol),[15] whereas for germanium, data are available only for Ge^0 ($-15.7\cdot10^{-6}$ emu/mol)[16] and Ge^{4+} ($-7\cdot10^{-6}$ emu/mol)[14]. Using these data, one can estimate a value of at least $-140\cdot10^{-6}$ emu/mol for $Ca_6[Ge_2N_6]$, however, in our opinion this overestimates the real value since no covalent bonds are taken into account.

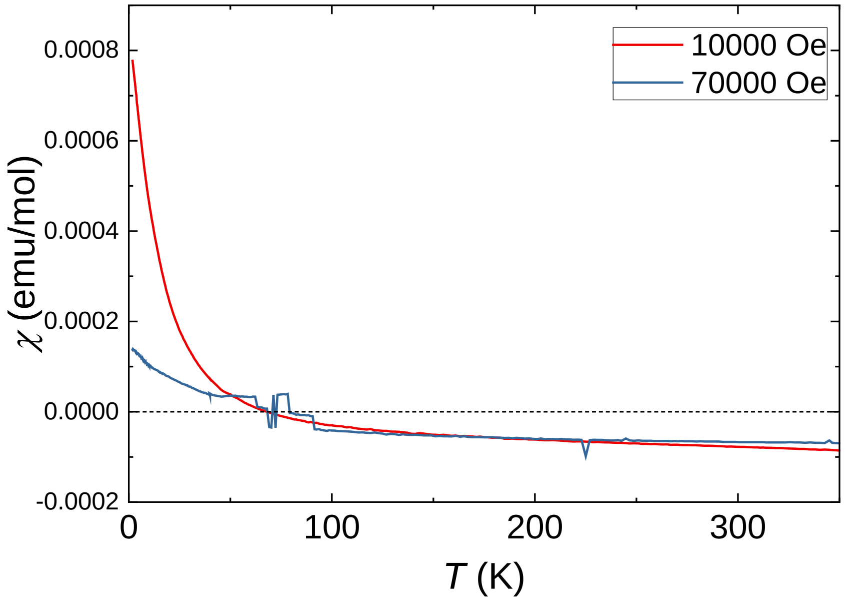

Figure S2. Molar magnetic susceptibility, only 10000 Oe and 70000 Oe shown. Considering the high-temperature part, it is obvious that $Ca_6[Ge_2N_6]$ is diamagnetic.

Electronic conductivity investigations

Electrical resistivity measurements were performed on the $Ca_6[Ge_2N_6]$ powder in the temperature range from ≈150 K to 300 K with a sapphire pressure cell inside a helium flow cryostat at zero magnetic field. The measurement set-up was a van der Pauw four-terminal configuration using a Keithley DC current source and a Hewlett Packard nanovoltmeter. As the cryostat is attached to the glovebox, all sample handling could be done in Ar atmosphere.

The measured electrical resistivity in fig. S2 shows activated semiconducting-like behavior with a band gap of ~0.28 eV, as compared to value of 1.05 eV calculated from the electronic structure. However, repeated measurements of the same sample showed a peculiar rise of resistivity (by an order of magnitude) over previous experiments. There are several possible explanations for the increase of resistivity ranging from gradual degradation of the small contact points/current paths between the grains to the degradation of the sample via oxidation, or a reaction with residual nitrogen in the glovebox. As no change of powder X-ray diffraction diagrams after the resistivity measurements could be observed, we can conclude that a bulk change of the sample did not occur. The reactions with oxygen or nitrogen also cannot be the only culprit as the resistivity was observed to also gradually increase after the actual resistivity measurements while the cryostat was still sealed off from the atmosphere of the glovebox (without the turbopump running though). Without a large amount of further experimentation, it is impossible to completely understand the measured resistivity of $Ca_6[Ge_2N_6]$, so the authors cannot unconditionally guarantee that the measured resistivity dependence should be attributed to the bulk of the sample.

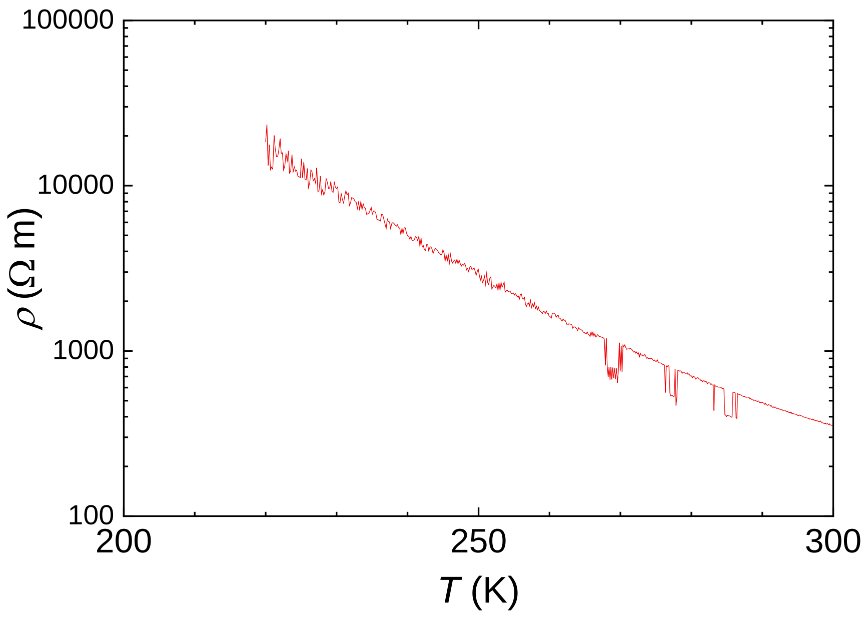

Figure S3. Electrical resistivity of $Ca_6[Ge_2N_6]$ as measured in a sapphire pressure cell. See text for discussion. Below 220 K, the resistivity is not shown as it becomes too large to be measured properly with our experimental set-up.

Electronic structure calculations

The electronic structure calculations were performed by using the all electron, local orbital full-potential method (FPLO) within the local density approximation.[17] The Perdew-Wang parametrization was employed.[18] The first Brillouin zone was sampled with a mesh of 20 x 20 x 20 and the linear tetrahedron method was applied to evaluate the Brillouin zone integrals. The experimentally determined crystal structure data (see the supporting information) were used in the calculations. Real-space chemical bonding analysis based on the electron localizability approach combining electron density (ED) and electron localizability indicator (ELI) were carried out.[19-21] The ED and ELI were calculated by a module implemented in the FPLO method.[22] Topological analysis of the ED and the ELI using the quantum theory of atoms in molecules (QTAIM) was realized by the program DGrid.[23-24] The number of atoms contributing to a bond was determined by applying the basin intersection technique.[25]

References

[1] S.J. Clarke, G.R. Kowach, F.J. DiSalvo, *Inorg. Chem.* **1996**, *35*, 7009-7012.
[2] S.C. Junggeburth, O. Oeckler, W. Schnick, *Z. Anorg. Allg. Chem.* **2008**, *634*, 1309-1311.
[3] S.C. Junggeburth, O. Oeckler, D. Johrendt, W. Schnick, *Inorg. Chem.* **2008**, *47*, 12018-12023.
[4] S.J. Clarke, F.J. DiSalvo, *Inorg. Chem.* **2000**, *39*, 2631-2634.
[5] M.I. Baraton, R. Marchand, P. Quintard, *J. Mol. Struct.* **1986**, *143*, 9-12.
[6] I.G. Nakhutsrishvili, D.N. Mogilyanskii, D.A. Dzhishiashvili, *Inorg. Mater.* **1994**, *30*, 1401-1402.
[7] V. Petříček, M. Dušek, L. Palatinus, *Z. Kristallogr.* **2014**, *229*, 345-352.
[8] G.M. Sheldrick, *Acta Crystallogr. A* **2008**, *64*, 112-122.
[9] G.M. Sheldrick, *Acta Crystallogr. C* **2015**, *71*, 3-8.
[10] Crystal Impact - Dr. H. Putz & Dr. K. Brandenburg GbR, *Diamond - Crystal and Molecular Structure Visualization*, Kreuzherrenstr. 102, 53227 Bonn, Germany, **2017**.
[11] K. Honda, *Ann. Phys.* **1910**, *337*, 1027-1063.
[12] M. Owen, *Ann. Phys.* **1912**, *342*, 657-699.
[13] P.W. Selwood, *Magnetochemistry, 2nd Ed.*, Interscience, New York, **1956**.
[14] G.A. Bain, J.F. Berry, *J. Chem. Educ.* **2008**, *85*, 532-536.
[15] S. Leoni, R. Niewa, L.G. Aksel'rud, Yu. Prots, W. Schnelle, T. Göksu, M. Cetinkol, M. Somer, R. Kniep, *Z. Anorg. Allg. Chem.* **2005**, *631*, 1818-1824.
[16] S. Hudgens, M. Kastner, H. Fritzsche, *Phys. Rev. Lett.* **1974**, *33*, 1552-1555.
[17] K. Koepernik, H. Eschrig, *Phys. Rev. B* **1999**, *59*, 1743-1757.
[18] J.P. Perdew, Y. Wang, *Phys. Rev. B* **1992**, *45*, 13244-13249.
[19] M. Kohout, F.R. Wagner, Y. Grin, *Int. J. Quantum Chem.* **2006**, *106*, 1499-1507.
[20] M. Kohout, *Faraday Discuss.* **2007**, *135*, 43-54.
[21] M. Kohout, *Int. J. Quantum Chem.* **2004**, *97*, 651-658.
[22] A. Ormeci, H. Rosner, F.R. Wagner, M. Kohout, Yu. Grin, *J. Phys. Chem. A* **2006**, *110*, 1100-1105.
[23] R.F.W. Bader, *Atoms in Molecules - A Quantum Theory*, Clarendon Press, Oxford, **2003**.
[24] M. Kohout, *DGRID*, 4.6, Radebeul, Germany, **2015**.
[25] S. Raub, G. Jansen, *Theor. Chem. Acc.* **2001**, *106*, 223-232.

Author Contributions

L. Link grew the single crystals and analyzed single crystal X-Ray diffraction data of $Sr_6[Ge_2N_6]$ and wrote the original draft, M. Pathak prepared single-phase powder samples of $Ca_6[Ge_2N_6]$, $Ca_2[GeN_2]$, $Ca_4[GeN_4]$, $Sr_7[GeN_4]N_2$, and Ge_3N_4, P. Höhn prepared single-phase powder samples of $Ca_6[Ge_2N_6]$ and $Sr_2[GeN_2]$, F. Jach analyzed the powder X-Ray diffraction data and collected and interpreted IR and Raman data. P. Koželj performed the magnetization and electronic conductivity measurements and interpreted the data, A. Ormeci performed the electronic structure calculations, and P. Höhn and R. Niewa wrote the paper with the help of all authors, performed reviewing and editing.
